# Impact and Lessons Learned from a National Consortium for Participatory Health Research: PartKommPlus—German Research Consortium for Healthy Communities (2015–2018)

**DOI:** 10.1155/2018/5184316

**Published:** 2018-09-17

**Authors:** Michael T. Wright, Susanne Hartung, Mario Bach, Sven Brandes, Birte Gebhardt, Susanne Jordan, Ina Schaefer, Petra Wihofszky

**Affiliations:** ^1^Catholic University of Applied Sciences Berlin, Institute for Social Health, Köpenicker Allee 39-57, 10318 Berlin, Germany; ^2^Robert Koch Institute, Department of Epidemiology and Health Monitoring, General-Pape-Straße 62-66, 12101 Berlin, Germany; ^3^Lower Saxony Association for Health Promotion and Academy for Social Medicine, Fenskeweg 2, 30165 Hannover, Germany; ^4^Alice Salomon University of Applied Sciences, Alice-Salomon-Platz 5, 12627 Berlin, Germany; ^5^Esslingen University of Applied Sciences, Department of Social Work, Health, and Nursing, Flandernstraße 101, 73732 Esslingen a. Neckar, Germany

## Abstract

Integrated strategies of health promotion at the municipal level are receiving particular attention in public health policy and practice in Germany. These strategies are intended to provide a coordinated approach to health promotion during the entire lifespan, with a particular focus on vulnerable communities. They are also intended to be participatory in both their design and implementation, involving all sectors of the social welfare, educational and healthcare systems, civil society, and the general public. PartKommPlus—German Research Consortium for Healthy Communities is examining such strategies using participatory forms of research. The goal is to determine how participation can best be planned and implemented and what effects this participation has. In this article the work of PartKommPlus from the first funding phase (2015–2018) will be described with particular attention to the lessons learned and the forms of impact which are being considered as part of the participatory research process.

## 1. Background

Under the coordination of the Federal Center for Health Education in Germany (BZgA) and EuroHealthNet, the European Union initiative “Closing the Gap: Strategies for Tackling Health Inequalities in Europe” was launched in 2004 to bring together the evidence regarding the causes of health inequality and the strategies to address them [1, 2]. A central recommendation for the member states is the development of an appropriate and effective national strategy [3]. A cornerstone of the German response has been the Cooperation Network “Equity In Health” (EIH), a national initiative founded in 2003 by the BZgA. An important goal of EIH is promoting the establishment of integrated municipal strategies (IMS) for health promotion, also known as “chains of prevention” [4–6]. These strategies are intended to provide a coordinated approach to health promotion during the entire lifespan, with a particular focus on vulnerable communities. The IMS aim to involve all sectors of the social welfare, educational and healthcare systems, civil society, and the general public in designing long-term strategies to improve and maintain the health of the population [7]. Local stakeholders are supported by coordinators located at the regional Association for Health Promotion (AHP) or a similar body found in each of the sixteen states in Germany. The new Law on Prevention requires all states to develop health promotion and prevention strategies while providing new funding mechanisms for these strategies. This includes supporting the AHP as they assist municipalities in setting up and maintaining IMS [8].

The need to establish IMS as an integral part of population-based health promotion strategies was identified more than twenty years ago. The concept was first taken up by the Healthy Cities movement which was formed in Europe to implement the WHO Ottawa Charter on Health Promotion. The Healthy Cities Network now comprises over 1,200 municipalities worldwide with over 70 in Germany. Research on the impact of Healthy Cities has been limited, largely due a lack of consensus on suitable indicators and their measurement [9]. This controversy relates to the current international discussion on the need to develop meaningful ways to evaluate the effects of complex health promotion strategies [10, 11]. An evaluation of the Healthy Cities Network in Germany [12] revealed progress in most cities regarding the goals of the IMS; however, 90% of those responding reported structural and organizational factors which significantly hindered such strategies. These included inadequate resources; inadequate understanding of the Healthy City concept; lack of commitment to the program of action proposed by Healthy Cities; inadequate integration into the national network and into the local political process; and inadequate documentation and evaluation. The latter is often characterized by a lack of clarity regarding the overall strategy and the intended objectives. Positive outcomes were more likely in larger cities with a longer history of involvement in the network and with continuity in local program coordination.

Another body of evidence which can inform the implementation of IMS is the literature regarding the building of local coalitions for the purpose of improving the health of a specific group of people (most commonly in a neighborhood), often focusing on a specific health problem or set of health problems. Largely originating in North America, this body of work investigates the factors supporting and hindering such coalitions, formulating the findings in terms of principles to guide the practice of coalition-building and maintenance [13–19].

There is an emergent, comparable literature in Germany on coalitions, also with a strong focus on specific neighborhoods or districts in a city. In this literature two priority issues have been identified as needing further attention: community engagement, particularly the engagement of vulnerable groups, and governance strategies [20]. A review of the German literature by Bär [21] revealed three emerging approaches for IMS: (1) top down, expert-centered—e.g., led by municipal governments [22]; (2) bottom-up, citizen-led—e.g., as found in broad-based community organizing [23]; and (3) participatory strategies initiated by professionals—e.g., as in the city of Flensburg [24]. According to Bär, the first approach appears to be most common in Germany, the second least common, and the third is receiving increasing attention.

## 2. The Research Consortium PartKommPlus

The focus of PartKommPlus—German Research Consortium for Healthy Communities is to study the process of building and maintaining IMS in Germany, with a particular focus on the issue of participation. Attention is being given to questions regarding how to establish ongoing, intersectoral cooperative structures for health promotion and to questions regarding the engagement of vulnerable communities in developing IMS. The knowledge gained will be applied to assist EIH and other interested parties in using participatory methods to establish IMS in more municipalities and to support an ongoing process of mutual learning to strengthen existing IMS.

The consortium was formed by members of the German Network for Participatory Health Research (PartNet) in response to a call of the Federal Ministry for Education and Research (BMBF) in the funding stream “Prevention Research” to build research consortia composed of research institutions, service providers, and local communities in order to answer key questions related to improving the infrastructure for health promotion and prevention, particularly for vulnerable communities. The subprojects of PartKommPlus were chosen based on pragmatic considerations (where could viable partnerships be formed) and the desire to maximize diversity in terms of geographical distribution in the country, the topics and communities to be addressed, and the participatory methods to be employed. PartKommPlus is one of seven research consortia being funded by the Ministry. The funder has exercised no influence in terms of focus or content of the work, explicitly supporting innovation and experimentation regarding participatory process and methods. This has included providing monetary support for various cooperative structures, based on the individual needs of the local projects and allowing the projects and the coordinating institution of the consortium to make changes, based on their emergent needs. This high level of flexibility on the part of the funder and the explicit support for the often unpredictable participatory processes has given the consortium the basis required by participatory research which is often not provided [25].

PartKommPlus is based on the internationally recognized principles of participatory health research (PHR) as formulated by the International Collaboration for Participatory Health Research (ICPHR). PHR is an umbrella term for the various local and regional traditions among the over twenty countries represented in the ICPHR, like* community-based participatory research*,* participatory action research*,* interactive research*,* participatory rural appraisal*,* militant research*, etc. The ICPHR has defined the core, defining principle of PHR as maximizing the participation of those whose life or work is the subject of the research in all stages of the research process, from the formulation of the research question and goal to the dissemination of the findings [26]. By engaging the people who are the subject of the research in the research process itself, data can be generated which have an immediate relevance for understanding local problems and for developing local solutions to the problems identified, in the case of PartKommPlus, integrated local strategies for health promotion. PHR also addresses the ethical imperative of people being directly involved in decisions which affect their health and well-being [27].

PartKommPlus is unique in two ways. It is the largest participatory research project funded in Germany to date, a country in which participatory forms of research have been rare [28, 29]. The size and structure of PartKommPlus also appears to be a distinguishing characteristic, no comparable consortium having been found in our search of the literature.

This article describes lessons learned and how impact has been conceptualized at the consortium level during the first phase of funding (2015–2018). The article is based on working papers and observations produced over the course of the three years by the consortium members in the context of joint colloquia and coordinating meetings. Various constellations of local and national partners participate in the meetings (see below).

## 3. Structure and Goals of PartKommPlus

PartKommPlus is providing new knowledge on the role of participation in municipal health promotion strategies. In line with the international discussion, we define participation as not just taking part, but as having influence on central aspects of one's living and work environments [27, 30, 31]. We are focusing specifically on the following issues which are raised by participation in municipal health strategies:

- *Cooperation and coordination (governance)*: local authorities are challenged to provide a form of oversight which is based on cooperation and consensus and which coordinates vertically between levels (administrative level, intermediate level, district level, and project level) as well as horizontally between the various functional departments and disciplines. This presumably requires specific forms of governance which we want to describe.
- *Forms of participation*: the various stakeholders—local authorities, civil society, social service, and health providers, and local citizens—can influence municipal strategies in different ways. We are looking at the various forms which this influence can take.
- *Impact of participation*: the various forms of participation on the part of the stakeholders can presumably have different effects on the municipal strategies, for example, in terms of the focus of the strategy, the measures taken at the local level, and their outcomes. We seek to describe these impacts.
- *Role of participatory health research*: the consortium is applying various forms of participatory research to support the development and maintenance of municipal strategies. We are looking at how this form of research can contribute to the work at the local level.
- *Participatory epidemiology and health reporting (surveillance and monitoring)*: data on the health of the population is a basis for all public health activities. Here we are looking at how participatory forms of data collection and analysis can support municipal and regional strategies in their work.
- *Participation within a research consortium*: participatory research projects are commonly local in scope. Within PartKommPlus we seek to bring together the knowledge gained from local studies to contribute to a national strategy. To do this, we are developing ways to work together in a participatory fashion and are reflecting specifically on our own participatory process.

PartKommPlus is composed of seven subprojects (descriptions of the individual projects can be found at www.partkommplus.de). Each project has its own specific questions and goals which are related to the above themes. Five of the projects are based at the local level, with research studies taking place in eight different communities located in six different states (Baden-Württemberg, Berlin, Brandenburg, Hamburg, Hesse, and Lower Saxony (Berlin and Hamburg are so-called city-states in which the city constitutes its own state jurisdiction)). Two of the projects are focused on general issues related to participatory processes in municipal health promotion. One of the projects is focusing on issues of governance in IMS, while the second project is developing participatory forms of health reporting and epidemiology (see Table 1). The consortium is coordinated by the Catholic University of Applied Sciences Berlin (KHSB). The Berlin-Brandenburg Association for Health Promotion (GBB) is in charge of an important part of the communication strategy for the consortium, which consists largely in interacting with the larger IMS community through a dedicated Internet site called* inforo* (www.inforo-online.de).

## 4. Participatory Process in PartKommPlus

At the heart of each of the seven subprojects is a participatory research process involving stakeholders at various levels, including local community members. The design of the projects varies widely, however, and each project has changed over time. These changes have included trying out different forms of engagement and adapting the research questions and methods to the differing interests of a growing group of stakeholders. The projects have also focused their work by using different approaches to participatory research, such as Appreciative Inquiry [32], peer research [33], participatory evaluation [34], and participatory epidemiology and health reporting [35]. Each approach has a specific frame of reference which sets a specific focus in terms of the participatory process.

The consortium as a whole has also sought to conduct its work in a participatory way. Given the lack of documented participatory research consortia of this size, it has been a process of experimentation to find forms of working together which not only maximize the participation of each lead institution in decision-making at the consortium level, but which also bring the voices of local people into the work of the consortium as a whole. The latter has proven to be the larger of the two challenges, but also the most rewarding in terms of the mutual learning process.

We began with a relatively conventional structure with a coordinating body in charge of integrating and synthesizing the data from the five projects taking place at the local level. The data collection was intended to take place at colloquia scheduled twice a year, each lasting three days and focusing on a topic related to the overarching research questions and being attended by representatives from each of the five local projects. In addition, interviews were to be conducted with people taking part in the five projects and other local data were to be gathered so as to address questions of governance and monitoring. The idea was to draw together systematic data from each of the five subprojects and to discuss these data at the colloquia so as to answer the overarching research questions of the consortium.

This plan did not work, for two primary reasons. Firstly, the five representatives of these projects felt that they were being made the objects of researchers from outside of the local context. They felt that they were being called to deliver data, but that they did not have sufficient control over what data were collected or how they would be analyzed. Several attempts were made to be more transparent about the central data collecting process and to include the five projects in that process, until we concluded that the problem was a structural one. We recognized a parallel process taking place in the local municipalities. Just as the people at the local level initially felt like they were the objects of research from the lead institutions, so too did the lead institutions feel like they were the objects of research from the consortium leadership. We were surprised by this dynamic because all the consortium partners were involved in designing the initial structure for the integration and synthesis of the data. It seems that a negotiation of power and control needs to take place at the start of any participatory research project, regardless of the time spent in collaborating on an initial plan. Apparently, this principle does not only apply to community members at the local level without prior research experience, but also to experienced researchers in a consortium structure, if they are not satisfied with the amount of influence they are having on the research process.

Secondly, participatory research is at its heart a local process [26]. In PartKommPlus the research processes at the municipal level are focused on maximizing the participation of the various stakeholders thereby enabling a broad ownership of the local projects. Building trust and ownership at the local level stands in contrast to a central and, for many partners, abstract and geographically remote process of data synthesis.

Over the years of the funding phase we decentralized our data collection and analysis strategy at the consortium level, thus departing from our initial structure. This bottom-up, inductive approach has led to identifying topics in which different constellations of lead institutions and stakeholders from the various projects bring together what they have learned about a topic of common interest. And each of these subgroups is deciding how they will work together and what product they will produce. For example, a group of peer researchers may come together to produce a list of criteria for participating in research. Whereas, a group of academic researchers may write a journal article on how participatory health research differs methodologically from other forms of health promotion research. Or a group of practitioners and academic researchers may design a tool for helping municipalities in setting up local strategies. Thus, not all subprojects are involved in all topics, and the option is also available for a subproject to work on a specific topic which only applies to its focus area. The result will not be a neat data synthesis from which answers to all research questions can be formulated, but rather a diverse assortment of various types of knowledge, products, and forms of reflection and analysis regarding municipal strategies of health promotion and participation.

We have continued meeting twice a year at the colloquia, but the focus has shifted to mutual support and exchange of ideas, for example, by sharing lessons learned at the local level and through peer supervision. The subprojects have also taken on an increasing role in determining the focus and structure of the colloquia, including an increasing involvement of their local partners.

## 5. Identifying the Types of Impact in PartKommPlus

As our work progresses, we are turning our attention increasingly to issues of data analysis, including that of impact. Here we present an initial description of the types of impact taking place in our work.

An important emerging issue internationally, regardless of the participatory research approach, is how best to report on research impact. A narrative approach is increasingly promoted as being most valuable in contexts of applied research [36]. A simple listing of impacts, however thorough in the description, fails to address the ways in which the impacts interact and how various factors determine how impacts emerge or are stymied. A narrative makes these connections and is thus instructive for those wanting to apply the findings to their own context. PartKommPlus will be using impact narratives at the project and consortium levels in order to provide a more coherent and comprehensive picture of the evidence.

The impact of health promotion can be described in terms of various outcomes. Bauman and Nutbeam [37] draw a distinction between health promotion outcomes (e.g., social action and influence), intermediate health outcomes (either program impact or short-term outcomes, e.g., an effective preventive health service), and social health outcomes (long-term outcomes, e.g., like quality of life). Thus, from a health promotion perspective, it is important to differentiate between the project level and higher structural levels, with different time scales for the various levels.

A central concern of PHR is impact in a broad sense. As a form of action research, PHR has the explicit intention of bringing about social change. In PHR learning and research are not considered separate entities. Social learning (learning together and from each other) is a fundamental dimension of the PHR process and the continual cycle of “look, reflect, act” underpins the dynamics of developing a connected knowing [38]. This means trying to understand the other person or idea through dialogue from relations of trust and empathy [39]. Everyone learns as a coresearcher to differing degrees. Ideally, the process should engage the participants in transformative learning, i.e., changes in the way they see the world and themselves [40, 41], through interactive processes which address both the personal and the collective. In turn, this generates an intention of being able to act based on the research findings, thus having a wider impact beyond the scientific community in the narrow sense. On the whole, how social change is defined is largely determined by whether the approach is pragmatic (that is, focused on issues of practical utilization) or emancipatory (where the focus is on changing the way people think and act in their world)—or an attempted combination of both [42, 43].

The work of Cook et al. [44] has demonstrated the difficulty authors have in recognizing and articulating impact in PHR. This includes recognizing the impact of participation on the research process and capturing the longitudinal aspect of impacts that occur long after a project has been completed. In an extensive review of the English language literature, Jagosh et al. [45] identified, selected, and appraised a large-variety sample of primary studies describing PHR partnerships. They used key realist review concepts to analyze and synthesize the data, employing the PHR partnership as the main unit of analysis (compare Jagosh et al. [46]). The link between the participatory research process and the outcomes in these partnerships was explained using the middle-range theory of* partnership synergy*, which demonstrates how PHR can (1) ensure culturally and logistically appropriate research; (2) enhance recruitment capacity; (3) generate professional capacity and competence in stakeholder groups; (4) result in productive conflicts followed by useful negotiation; (5) increase the quality of outputs and outcomes over time; (6) increase the sustainability of project goals beyond funded time frames and during gaps in external funding; and (7) create system changes and new unanticipated projects and activities.

A review by Staley [47] suggests an interesting, preliminary typology describing positive and negative impacts which can result from PHR, based on an extensive review of published and gray literature on the INVOLVE strategy for public involvement in the research of the National Health Service in the UK. This includes impact on the research process (agenda, design, delivery, and ethics), impact on the public involved, impact on academic researchers, impact on other research participants, impact on the wider community, impact on community organizations, and impact on change processes (e.g., improved service delivery). This typology is particularly useful for characterizing the work of PartKommPlus as it takes into account both impacts at the project level and at higher structural levels.

We present here an initial typology to describe the impact of our work which we will begin to apply over the course of the coming year. Following Staley, it will be important to describe both the positive and negative impacts/challenges which PartKommPlus has had.

### 5.1. Impact on the Participants

Each of the seven research projects is being conducted in a partnership between different constellations of academic institutions, community organizations, professionals in the healthcare, social service and education systems, and engaged citizens. The workings of the consortium as a whole, as organized by the coordinating institution, can be considered an additional level which participants should consider in terms of how PartKommPlus has impacted them.

In order to understand the impact in each project and at the consortium level it will be necessary to describe the specific partner constellation and the degree of participation each partner has in the project. As stated above, participation is defined as the degree of influence a partner has had on the research process. This includes partners at all project levels, from the project leads to those who have provided information about their lives or work over the course of the project, and includes both academic researchers and the members of research teams without formal research training. Expected impacts, based on existing findings in participatory research projects, include insights into the perspectives and needs of other stakeholders, empowerment, learning new forms of research practice, gaining research skills, gaining interpersonal skills regarding facilitation and negotiation, insights into one's own workplace or living situation, new ideas for health promotion in a specific context or for a specific group of people, and a growth in self-confidence. Impact at the participant level can be assessed using the data gathered through participatory methods (such as photovoice or community mapping) and over the course of conversations, reflection exercises, and other forms of interaction with those who have been involved in the projects.

### 5.2. Impact on the Municipalities

The intention of PartKommPlus is to have an impact on how local municipal strategies are planned and implemented, with a particular focus on issues of participation, as detailed in the research goals above. This includes improved communication between the various stakeholders; a new awareness of what is necessary in order for participation to take place, in terms of resources, structures, and ways of working; and a new common understanding among the stakeholders for reporting, planning, and action.

The seven subprojects are imbedded in different ways in local, regional, and/or national contexts. It will be important to describe in what way each of the projects is imbedded and what effects were intended by the various stakeholders. This description will provide a basis not only for depicting the specific impacts reached or not reached, but also for explaining how and why such impacts were made possible or were not successful.

Evidence for impact on local municipal strategies can be obtained from the partners involved in the research projects and from those with whom the projects have interacted in order to effect change. Other sources for mapping impact are municipal reports on health or social issues in which public authorities, public health practitioners, activists, and other citizens can be involved.

### 5.3. Impact on the IMS Community

An explicit goal of the Ministry for Education and Research (BMBF) in funding research consortia is to generate new knowledge which can directly support the development of more effective structures for prevention and health promotion in Germany. As described above, PartKommPlus has positioned itself strategically in order to contribute to the establishment and sustainability of integrated municipal strategies for health promotion by focusing on the issue of participation. To what degree PartKommPlus has a unique and specific impact on the larger discourse and practice regarding IMS in the country will be difficult to assess. Evidence can be gathered through the national website inforo dedicated to IMS (see above) through which the work of PartKommPlus is being disseminated. The site provides several different opportunities for interaction between the consortium and the wider community of practice. Other evidence can be obtained by documenting how the work of the consortium as a whole and that of the subprojects is being discussed in various local, regional, and national contexts, for example, at conferences, in planning forums, in the media, and in professional and academic publications. It can also be observed to what extent the consortium has widened the circle of municipalities taking part in our colloquia and other forums.

### 5.4. Impact on the Research Community

PartKommPlus is the largest PHR project to date in Germany. We are seeking specifically to establish PHR within the landscape of health promotion research in a country in which participatory forms of research are relatively uncommon. It will be difficult to ascertain the unique impact of PartKommPlus on the scientific community, given the growing number of projects and initiatives promoting participatory research in Germany, and given the various ways in which the German Network for Participatory Research (PartNet) is involved in addressing issues of research practice and policy. However, evidence for impact can be gathered in terms of how and where the work of PartKommPlus as a whole and the work of the subprojects are being cited in research contexts, including both conferences and publications, and the degree to which we are collaborating with other researchers.

## 6. Summary of Lessons Learned

- We needed to experiment with different formats and means of communication to achieve a level of participation at the consortium level which meets the expectations of the local partners.
- In spite of longstanding working relationships between many of the consortium members and the mutual process of writing the grant proposal, we needed to negotiate power and control at the consortium level and build mutual trust.
- There is a parallel process operating at the local and the consortium levels. At both levels, participation, ownership, and trust are being simultaneously negotiated.
- Maximizing the participation of each lead institution in the decision-making has been challenging, but not as challenging as bringing the voices of local people to the consortium level.
- The research processes at the municipal level are focused on maximizing the participation of the various stakeholders, thereby enabling a broad ownership of the local projects. Building trust and ownership at the local level stands in contrast to a central and, for many partners, abstract and geographically remote process of data synthesis.
- A decentralized, bottom-up, inductive approach to data analysis at the consortium level is more appropriate than applying a centralized data collection and analysis strategy. This approach means that we will not have a neat data synthesis from which answers to all research questions can be formulated. However, such an approach allows for a diverse assortment of various types of knowledge, products, and forms of reflection and analysis regarding municipal strategies of health promotion and participation. It also maximizes opportunities for the various stakeholders to take part in the analysis process.
- The consortium meetings have fulfilled the important function of providing mutual support and a place for sharing ideas and experiences, thus supporting local capacity building.
- The issue of impact needs to be conceptualized as multileveled, including the impact on the participants, the impact on the municipalities, the impact on the IMS community, and the impact on the research community.

## 7. Looking Forward

The size and scope of PartKommPlus provide a unique opportunity to observe the impact of PHR at several levels of health promotion research and practice in Germany. Given that a broad impact has been the explicit intention of PartKommPlus from the start, we are seeking to document various forms of change which have been made possible through our work. The strength of the evidence will vary, as commented above; however, the variety of sources, participants, methods, and settings will provide a strong basis for describing what impacts are possible and which factors promote and hinder these impacts. These findings will, in turn, provide a foundation for advancing IMS and PHR in Germany while serving as an example internationally for assessing impact of a national research consortium active in several regions of a country.

## Figures and Tables

**Table 1:** The projects in PartKommPlus.

Project Title (with abbreviation)	Focus
Parents Asking Parents: From Model Project to Municipal Roll-Out (ElfE^2^)	Parent peer research to promote the participation of vulnerable families in pre-schools

Participatory Evaluation of the Prevention Chain in Braunschweig (PEBS)	A participatory evaluation of the Braunschweig network to prevent poverty among families with children

Development of Municipal Health Promotion Strategies (KEG)	Developing municipal strategies for health promotion through a dialogue between research and practice

Health Promoting Neighborhoods (Age4Health)	Engaging vulnerable older people in developing local health promotion strategies

People with Intellectual Disabilities and Health Promotion Programs (HEALTH!)	Inclusion of people with intellectual disabilities in health promotion strategies

Municipalities and Health Insurance Funds—Cooperating for Healthy Local Environments (K^3^)	Governance in municipal health promotion strategies with the focus on the cooperation between health insurers and public authorities

Participatory Epidemiology: From Data to Recommendations (P&E)	Participatory approaches to epidemiology and health monitoring

## Data Availability

The data used to support the findings of this study are available from the corresponding author upon request.
